# Towards greenhouse cultivation of *Artemisia annua*: The application of LEDs in regulating plant growth and secondary metabolism

**DOI:** 10.3389/fpls.2022.1099713

**Published:** 2023-01-18

**Authors:** Ningyi Zhang, Haohong Yang, Tianqi Han, Hyoung Seok Kim, Leo F. M. Marcelis

**Affiliations:** ^1^ Horticulture and Product Physiology, Department of Plant Sciences, Wageningen University, Wageningen, Netherlands; ^2^ Smart Farm Convergence Research Center, Korea Institute of Science and Technology (KIST), Gangneung, Republic of Korea

**Keywords:** *Artemisia annua*, artemisinin, light spectrum, plant morphology, biomass, glandular trichome

## Abstract

Artemisinin is a sesquiterpene lactone produced in glandular trichomes of *Artemisia annua*, and is extensively used in the treatment of malaria. Growth and secondary metabolism of *A. annua* are strongly regulated by environmental conditions, causing unstable supply and quality of raw materials from field grown plants. This study aimed to bring *A. annua* into greenhouse cultivation and to increase artemisinin production by manipulating greenhouse light environment using LEDs. *A. annua* plants were grown in a greenhouse compartment for five weeks in vegetative stage with either supplemental photosynthetically active radiation (PAR) (blue, green, red or white) or supplemental radiation outside PAR wavelength (far-red, UV-B or both). The colour of supplemental PAR hardly affected plant morphology and biomass, except that supplemental green decreased plant biomass by 15% (both fresh and dry mass) compared to supplemental white. Supplemental far-red increased final plant height by 23% whereas it decreased leaf area, plant fresh and dry weight by 30%, 17% and 7%, respectively, compared to the treatment without supplemental radiation. Supplemental UV-B decreased plant leaf area and dry weight (both by 7%). Interestingly, supplemental green and UV-B increased leaf glandular trichome density by 11% and 9%, respectively. However, concentrations of artemisinin, arteannuin B, dihydroartemisinic acid and artemisinic acid only exhibited marginal differences between the light treatments. There were no interactive effects of far-red and UV-B on plant biomass, morphology, trichome density and secondary metabolite concentrations. Our results illustrate the potential of applying light treatments in greenhouse production of *A. annua* to increase trichome density in vegetative stage. However, the trade-off between light effects on plant growth and trichome initiation needs to be considered. Moreover, the underlying mechanisms of light spectrum regulation on artemisinin biosynthesis need further clarification to enhance artemisinin yield in greenhouse production of *A. annua*.

## 1 Introduction

Artemisinin is a sesquiterpene lactone produced in glandular trichomes of *Artemisia annua*, and is widely used in anti-malaria therapeutics (Greenwood and Mutabingwa, 2002). Artemisinin and its derivates are also found to be effective in treatments of several cancers and inflammatory and viral diseases (Efferth, 2007; Efferth et al., 2008; Ho et al., 2014), and may also be used in treatment at the early stage of mild-moderate COVID-19 (review in Uckun et al., 2021). Currently, plant material is the most important and almost the only source for extracting artemisinin (Ferreira et al., 2018; Shen et al., 2018). Given the low artemisinin content in *A. annua* plants (0.01% to 1%, on dry weight basis, Liu et al., 2006), many studies focused on elucidating the artemisinin biosynthesis pathway and the mechanisms underlying the transcriptional regulations of the genes involved in this pathway (review in Hassani et al., 2020). Recently, *A. annua* transgenic lines were reported to have high artemisinin content up to 3.2% of dry weight (Shen et al., 2018). Alternatively, artemisinin can also be produced using semi-synthetic approach (e.g. yeast), or by other crops engineered with genes involved in artemisinin biosynthesis. However, the semi-synthetic approach *via* yeast is only able to produce artemisinic acid, which needs to be further converted to artemisinin, resulting in a high cost for producing artemisinin *via* yeast (Peplow, 2016). The artemisinin content in transgenic plants of other species (e.g. tobacco) is even lower than artemisinin content in *A. annua* plants (Farhi et al., 2011). Moreover, plant-based delivery of artemisinin (e.g. using dried leaves or plants, or as a tea infusion) is more effective than using a comparable dose of pure artemisinin and can overcome resistance to pure artemisinin (Suberu et al., 2013; Elfawal et al., 2015; Daddy et al., 2017). Therefore, the demand for *A. annua* plant materials is rather high.

*A. annua* plants are generally cultivated in the field or directly collected from the wild (Ferreira et al., 2005). The environmental fluctuations (e.g light, temperature, water and nutrient levels) in the field strongly affect plant growth and artemisinin biosynthesis in *A. annua*. For example, the seasonal variation (during summer and autumn) of artemisinin content in field-grown plants fluctuates between 0.2% to 0.9% (Ferreira et al., 2018). Seasonal variations in plant growth and artemisinin content cause problems such as unstable supply of raw plant materials, unstable quality and quantity of secondary metabolites, and fluctuations in the market price. Furthermore, collecting plant materials from the wild threatens the survival of wild species and biodiversity in specific regions. In contrast to the field environment, greenhouses provide more stable plant growth conditions given the precise climate control and water and nutrient supply. Such a production system is widely used in producing horticultural products to allow all-year-round production and to achieve stable yield and standardized product quality (Marcelis et al., 2019). For example, a Dutch tomato greenhouse produces a fresh yield of 60-70 kg m^-2^ for year-round production, and is appreciated for constant product quality and reliable delivery; this further helps to achieve a well-organised market, with ~85% of the production is sold *via* growers’ associations and ~90% is exported (Heuvelink, 2018). The success of horticultural crop production in greenhouses shows a great potential in such an indoor plant production system for cultivating *A. annua* to achieve continuous supply of raw plant materials, stable product quality, and a less fluctuating market price.

In greenhouse production, supplemental lighting (e.g. LEDs) is frequently used as an additional light source when solar radiation is low in order to increase light intensity and to change light spectrum to improve yield and product quality (Kaiser et al., 2019; Affandi et al., 2020; Ji et al., 2020). Thus, supplemental LEDs also have potential to improve *A. annua* greenhouse cultivation. Light plays an important role in the activation of artemisinin biosynthesis. The expression of key artemisinin biosynthesis genes e.g. *AaADS*, *AaCYP71AV1*, *AaDBR2* and *AaALDH1* are strongly upregulated by light, leading to increased artemisinin biosynthesis (Hao et al., 2017; Zhang et al., 2018; Hao et al., 2019). Furthermore, red and blue light result in a higher artemisinin content compared with white light by enhancing the expression levels of several relevant genes (e.g. *AaHY5*, *AaWRKY9*, *AaDBR2* and *AaGSW1*) (Zhang et al., 2018; Hao et al., 2019; Fu et al., 2021). Additionally, ultraviolet-B (UV-B) radiation has been found to increase artemisinin content by upregulating the expressions of artemisinin biosynthesis genes and transcriptional factors (e.g. *AaADS*, *AaCYP71AV1*, *AaDBR2*, *AaCPR* and *AaMYB4*) (Rai et al., 2011; Pandey and Pandey-Rai, 2015; Ma et al., 2020; Li et al., 2021). Nevertheless, most studies focused on the light regulation of artemisinin biosynthesis. The effect of light conditions on *A. annua* growth, morphology and biomass production has been overlooked, yet these aspects are important in greenhouse *A. annua* production.

The objective of this study was to explore the potential of manipulating the light environment using LEDs to improve artemisinin biosynthesis. Furthermore, we aimed to elucidate whether supplemental LEDs leads to adverse (e.g. adding UV-B) or positive (e.g. adding red light) effects on plant biomass production. To this end, two experiments were conducted in a greenhouse, with the first experiment exploring the effects of adding supplemental red, blue, green and white light and the second investigating the interactive effects of UV-B and far-red radiation on plant biomass production, trichome formation and biosynthesis of artemisinin and its precursors.

## 2 Materials and methods

### 2.1 Plant material and growth conditions

Two experiments were conducted between January and July 2021 in a compartment (8 m × 8 m) of a Venlo-type glasshouse located in Wageningen, The Netherlands (52°N, 6°E). There were four rolling growth tables (1.7 m × 6.5 m) in the compartment. *A. annua* seeds (provided by Hortus Alkmaar, The Netherlands) were sown on the surface of potting soils filled in a plastic tray and then covered by vermiculite. When the first two true leaves were visible, individual seedlings were separated and transplanted to plastic pots (diameter = 19 cm) filled with potting soil. The plants were put on the rolling growth tables with a plant distance of 23 cm, resulting in a plant density of 19 plants m^-2^.

The photoperiod was 16 hours (from 4:00 to 20:00 hours). High-pressure sodium (HPS) lamps (600W, Philips, Eindhoven, The Netherlands) were used during light period when global radiation outside the greenhouse dropped below 150 W m^-2^ and were switched off when outside global radiation increased to values above 250 W m^-2^. Light intensity from the HPS lamps was on average 127 mmol m^-2^ s^-1^ at crop level (**Supplementary Figure S1**). The shading screen (Harmony 4215 O FR, Ludvig Svensson, Hellevoetsluis, The Netherlands) was closed when outside global radiation increased to values above 600 W m^-2^ and was opened when outside global radiation dropped below 500 W m^-2^. CO_2_ was kept at ambient. Setpoints of day and night temperature were 18 °C and 22 °C. Relative air humidity was set at 65%. Daily light integral of photosynthetically active radiation (PAR) from the sun, HPS lamps and the treatment LEDs, temperature and relative air humidity in the greenhouse compartment during the experiment are presented in **Supplementary Figure S2**.

### 2.2 Light treatments

Light treatments were started at transplanting the seedlings to pots and lasted for five weeks before the plants were harvested for destructive measurements. Two experiments were conducted, with each including four light treatments.

#### 2.2.1 Experiment 1

Four treatments were applied in Exp. 1 by adding LED modules that respectively provide supplemental red (Res Module, Philips. NL), green (Lumileds, NL), blue (Res Module, Philips, NL) and white (Res Module, Philips, NL) light in each treatment. In each plot, four LED modules were attached on a wooden frame such that the LED modules were distributed evenly in the plot. The LED frame was surrounded by a plastic film (with white colour facing the plot and black colour facing outside) with 20 cm depth from the frame top to minimize light treatments affecting each other (**Supplementary Figure S3**). The LED frame was kept at a distance of 50 cm from the plant canopy and the height of the frame was adjusted accordingly with the growing of plant height. The LEDs were kept on during the whole photoperiod (16 hours) and provided an irradiance of approximately 23 mmol m^-2^ s^-1^ at the canopy level. This irradiance was the maximum level reached by adding four green LED modules. For the other three treatments, part of the LED modules was covered by aluminium foil to lower the output to the same level as the green LEDs.

#### 2.2.2 Experiment 2

Four treatments were applied, including supplemental far-red, supplemental UV-B, supplemental far-red and UV-B and a control without any supplemental radiation (except for the radiation provided by supplemental HPS lamps that was received by all treatments). The LED modules were attached on a wooden frame and arranged in the same way as described in Exp. 1. In the control, a wooden frame without any LED modules was used to create a similar level of shading by the LED frames in the other three treatments (**Supplementary Figure S4**). In treatments in which supplemental far-red was used, four far-red modules (GreenPower far-red -production modules, Philips, NL) were attached on each frame, resulting a red (655-665 nm) to far-red (725-735 nm) ratio of approximately 0.3 at the plant level. The far-red modules were kept on during the whole 16-hour photoperiod. In treatments in which supplemental UV-B was used, one UV-B module (UV-B broadband lamps, Philips, NL) was attached on each frame, resulting in an intensity of 0.53 W m^-2^ at plant level. The UV-B lamps were turned on for 30 min daily (from 00:00 to 00:30 AM).

### 2.3 Measurements on light conditions

In Exp. 1, light spectrum of the LED modules used in each treatment was measured in darkness using a spectrometer (Type 3000, Apogee Instrument, USA) (**Supplementary Figure S5**). Distribution of PAR from the LEDs used in each treatments was measured by a quantum sensor (Li-250A Quantum meter, LI-COR, USA) (**Supplementary Figure S6**). Measurements were conducted in darkness with only the supplemental LEDs being turned on (i.e. no light from the sun and HPS lamps). In total 64 positions were measured in each plot, with a distance of 20 cm between each measurement spot.

In Exp. 2, distribution of the red to far-red ratio in each treatment was measured by a spectrometer (Type 3000, Apogee Instrument, USA) (**Supplementary Figure S7**). Measurements were conducted on 25 positions in each plot. Spectrum of the UV-B lamps was measured by a spectral-radiometer (BTS2048-UV-S, Gigahertz-Optik, Germany), and distribution of the UV-B light was measured at nine positions in a plot using ILT2400 (ILT, USA) (**Supplementary Figure S8**).

### 2.4 Non-destructive measurements

Five plants per plot were randomly chosen after two weeks from transplanting. Those plants were used to measure plant architectural traits twice per week, including plant height, leaf number and leaf length on the main stem. Plant height was measured from soil level to apex. Given that leaf senescence of the first two true leaves happened in early stage of plant development (usually before starting architectural measurements), the third true leaf was labelled and defined as the first leaf for measurements. Leaf length was measured as the distance along the midrib from the insertion point the leaf petiole on the main stem to the leaf tip. Leaf length was used to calculate leaf area non-destructively according to the relationship established from the destructive measurements (**Supplementary Figure S9**).

### 2.5 Destructive measurements

Plants used for destructive measurements in each plot were separated into three groups: three plants were used for trichome density measurement, six plants were sampled for measuring secondary metabolite content, and 15 plants were harvested by end of the experiment.

#### 2.5.1 Trichome density

Measurements were taken in week 3 (seedling stage) and week 5 (branching stage) after transplanting. For each measurement, three newly fully developed leaves were taken from each plant. A small part of the leaf was cut and put on a glass slide, followed by putting another glass slide on top of the leaf and gently pressing the top slide to make the leaf surface flat. Then the slides were put under a stereo microscope (MZ APO, Leica, Germany, with camera from Axiocam 305 color, Carl Zeiss, Germany) to take images from the adaxial leaf surface using a magnification of 8×. Trichomes in each image were counted and area of the leaf samples were analysed using ImageJ (National Institute of Health, Bethesda, MD, USA).

#### 2.5.2 Secondary metabolites

Plant samples were taken in week 3 and week 5, with three plants per plot being sampled each time. Leaves and stems were collected and frozen in liquid nitrogen, and then were separately stored in 20 ml tubes in -80°C freezer. Once all samples were collected, they were grinded (in the liquid nitrogen environment) and put in the freeze-dryer for five days at a temperature of -60°C and a pressure of 0.2 atm. Those freeze-dried samples were then used for measuring secondary metabolites including artemisinin, dihydroartemisinic acid, arteannuin B and artemisinic acid based on high performance liquid chromatography, following the protocols developed by Lapkin et al. (2009).

#### 2.5.3 Final harvest

In week 5, a total of 15 plants per plot, including the five plants used for non-destructive measurements, were harvested (exact harvest dates of different blocks and experiments were given in Supplementary Table S1). In addition to the architectural traits measured during non-destructive measurements, internode length and elevation angle of individual leaves on the main stem were measured on the day of final harvest. Internode length was measured as the distance between the insertion points of two successive leaves on the main stem. Leaf elevation angle was defined as the angle between the leaf midrib and the horizontal level. Then, the plant was sampled to measure leaf area from the main stem and side shoots separately, and fresh and dry weights of leaves and stem separately. Leaf area was measured by a leaf area meter (Li-3100, LICOR, Lincoln, NE, USA). Dry weight was measured after drying the samples for 72 hours at 70°C in an oven.

### 2.6 Statistical set-up and analysis

The experiments had a randomized block design, with four blocks for each experiment. 49 plants (7 × 7 plants) were grown in each plot, with the outer plants serving as border plants, resulting in a total of 25 experimental plants being used for measurements. The position of individual experimental plants were randomized weekly to avoid any possibly effects from the uneven light distribution. To optimize greenhouse space and labouring, the transplanting date (i.e. the start of light treatments) for each block in each experiment was spread during the whole experimental period (**Supplementary Table S1**).

Statistical analyses were conducted using R (http://www.r-project.org/). First, normality was tested using the Shapiro-Wilk test and homogeneity was tested using Levene’s test to determine whether residuals showed equal variances. For traits that did not show equal variance, log transformation of data was applied. For Exp. 1, differences between the four treatments were detected using one-way ANOVA (*p* < 0.05) with considering the block effects. When a significant difference was detected, a *post-hoc* test was conducted for pairwise comparisons between treatments, using Fisher’s Protected Least Significant Difference (LSD) test (*p* < 0.05). For Exp. 2, treatment effects were tested using two-way ANOVA (*p* < 0.05) with considering the block effects.

## 3 Results

### 3.1 Effects of supplemental radiation within and outside PAR on plant architectural development

**Figure 1:**
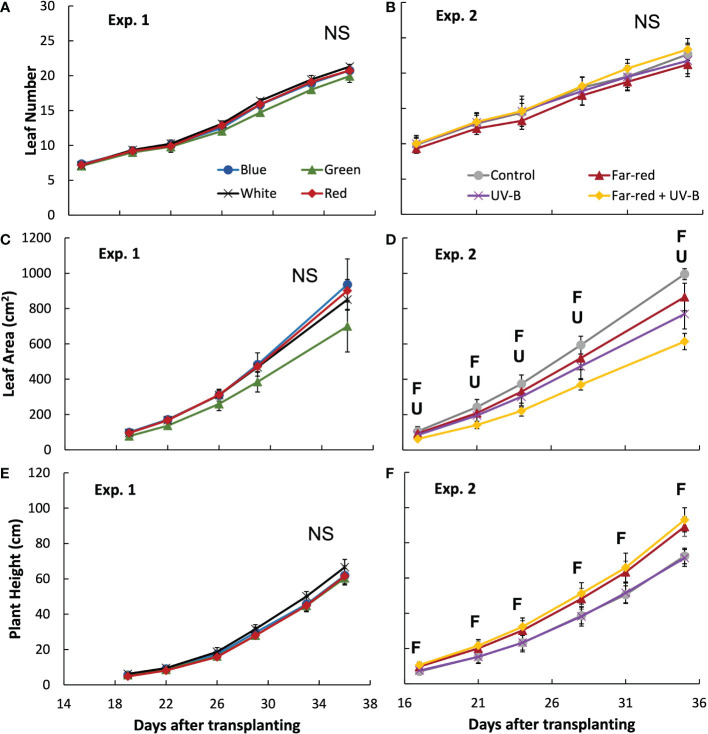
Leaf number **(A, B)**, leaf area on the main stem **(C, D)** and plant height **(E, F)** during the experiment in treatments of supplemental radiation within (A, C, E; Exp. 1) and outside (B, D, F; Exp 2) the range of photosynthetically active radiation (PAR) (values are mean ± s.e.; n = 4, with five plants in each statistical replicate). The supplemental radiation of different colours in panels A, C and E had an intensity of 23 mmol m^-2^ s^-1^. The control in panels B, D and F is treatment without supplemental radiation; supplemental far-red radiation resulted in a red to far-red ratio of 0.3 at the plant level, and supplemental UV-B had an intensity of 0.53 W m^-2^. “F” and “U” respectively indicate a significant effect of far-red and UV-B on a specific day after transplanting (*p* < 0.05). “NS” indicates non-significant effect was found.

**Figure 2:**
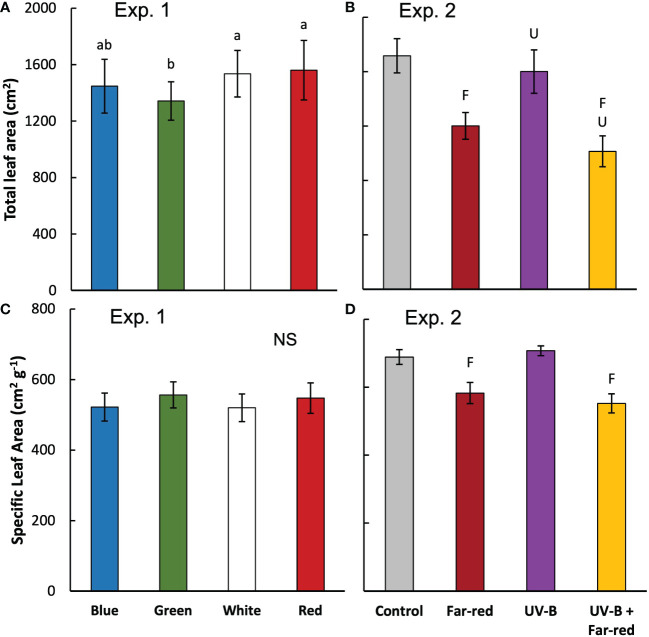
Total leaf area (from both main stem and side shoots) per plant **(A, B)** and specific leaf area (calculated as leaf area divided by leaf dry weight; average value from all leaves) **(C, D)** measured at final harvest in treatments of supplemental radiation within (A, C; Exp 1) and outside (B, D; Exp 2) the range of PAR (values are mean ± s.e.; n = 4, with 15 plants in each statistical replicate). Details on the treatment abbreviations can be found in Figure 1. In panel A and C, letters indicate significant differences (*p* < 0.05) and “NS” indicates non-significant difference. In panel (B and D, “F” and “U” respectively indicate a significant effect of far-red and UV-B (*p* < 0.05).

**Figure 3:**
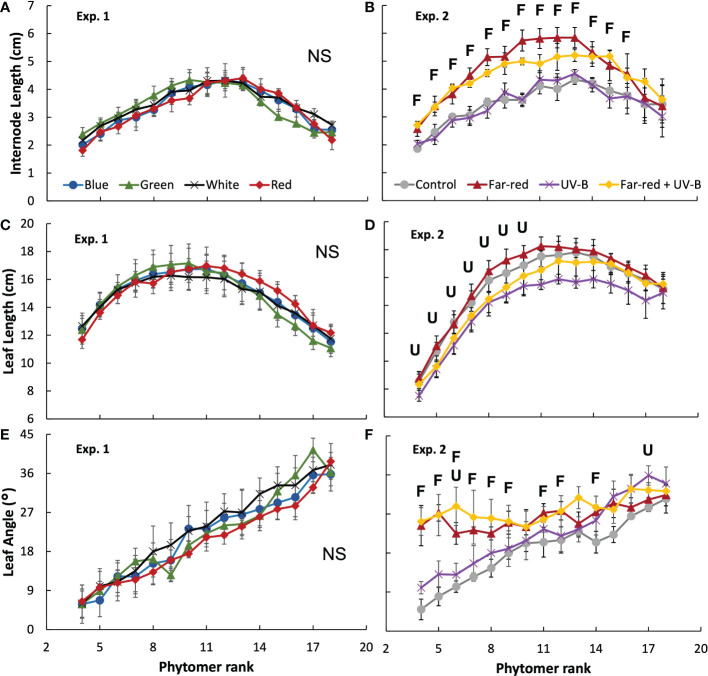
Internode length **(A, B)**, leaf length **(C, D)** and leaf elevation angle compared with horizontal **(E, F)** for each phytomer rank on the main stem at final harvest in treatments of supplemental radiation within (A, C, E; Exp. 1) and outside (B, D, F; Exp. 2) the range of PAR (values are mean ± s.e.; n = 4, with 15 plants in each statistical replicate). Details on the treatment abbreviations can be found in Figure 1. “F” and “U” respectively indicate a significant effect of far-red and UV-B on the traits measured at a specific rank (*p* < 0.05). “NS” indicates non-significant effects for all ranks.

The colour of supplemental radiation within PAR range (blue, green, red, or white) did not significantly affect plant architectural development. The time courses of leaf number, leaf area of main stem and plant height during the experimental period were hardly affected by the colour of the supplemental PAR (**Figure 1**). Supplemental green tended to slow down the development of leaf area but the effect was not significant (**Figure 1C**). At final harvest, total leaf area (including leaves from both the main stem and side branches) was significantly reduced by supplemental green compared with other colours of supplemental PAR (**Figure 2A**), whereas specific leaf area was hardly affected (**Figure 2C**). Final length of individual internodes and leaves on the main stem, as well as elevation angle (compared with horizontal) of each leaf, were not significantly affected by the colour of supplemental PAR (**Figures 3A, C, E**). The longest internodes and leaves appeared at the middle of the stem (**Figures 3A-C**), whereas leaf elevation angle kept increasing with leaf rank, indicating more flat leaves at the bottom of the plant and more steeper leaves at the top (**Figure 3E**).

Supplemental radiation outside PAR range (far-red and/or UV-B) had strong impacts on plant architectural traits, however, there were no significant interactive effects between far-red and UV-B on plant architecture. Both supplemental far-red and UV-B significantly reduced leaf area development during the experimental period, and lead to significant reductions in total leaf area at final harvest compared to control treatment without supplemental radiation (**Figures 1D**, **2B**). Specific leaf area was reduced by supplemental far-red but not affected by UV-B, whereas leaf number was not affected by either far-red or UV-B (**Figures 1B**, **2D**). Supplemental far-red significantly increased plant height during the whole experimental period, as well as internode length (**Figures 1F**, **3B**). Supplemental UV-B did not affect plant height nor internode length (**Figures 1F**, **3B**); however, it significantly reduced leaf length at the middle and bottom of the plant (**Figure 3D**). Supplemental far-red significantly increased elevation angle for nearly all leaves, leading to relatively evenly distributed leaf angle on the plant, whereas supplemental UV-B only had a marginal effect on leaf angle (**Figure 3F**).

### 3.2 Effects of supplemental radiation within and outside PAR on plant biomass

**Figure 4:**
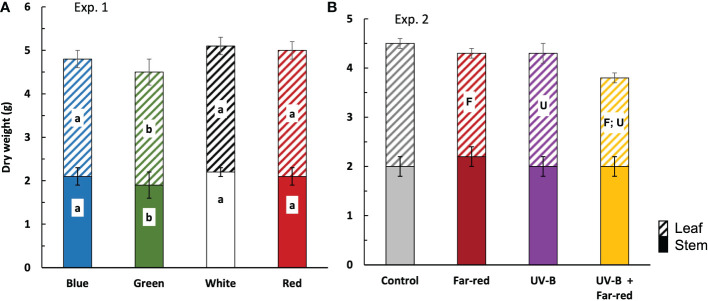
Plant dry weight at final harvest in treatments of supplemental radiation within (A, Exp. 1) and outside (B, Exp. 2) the range of PAR (values are mean ± s.e.; n = 4, with 15 plants in each statistical replicate). Solid bars are stem dry weight. Dashed bars are leaf dry weight. Details on the treatment abbreviations can be found in Figure 1. In panel **(A)**, letters indicate significant differences (*p* < 0.05). In panel **(B)**, “F” and “U” respectively indicate a significant effect of far-red and UV-B (*p* < 0.05).

In general, the colour of supplemental radiation within PAR range had smaller effects on plant biomass compared with the effects from supplemental far-red and UV-B. Supplemental green significantly reduced fresh and dry weights of both leaves and stem, compared with other colours of supplemental PAR, leading to lower plant fresh and dry weights (**Figure 4A**; **Supplementary Table S2**). However, plant fresh and dry weights from treatments with supplemental blue, red and white did not significantly differ from each other (**Figure 4A**; **Supplementary Table S2**). The colour of supplemental PAR only induced marginal effects on plant dry matter partitioning (~ 2% difference) (**Supplementary Table S2**).

There were no significant interactive effects between supplemental far-red and UV-B on plant fresh weight, dry weight, and dry matter partitioning. Both supplemental far-red and supplemental UV-B significantly reduced plant dry weight, which was due to the reductions in leaf dry weight but not stem dry weight (**Figure 4B**). Supplemental far-red significantly reduced fresh weights of leaves and stem, resulting in a lower plant fresh weight compared with treatments without supplemental far-red, whereas supplemental UV-B hardly affected plant fresh weight (**Supplementary Table S2**). Supplemental far-red significantly increased dry matter partitioning to stem (up to 8%) (**Figure 4B**; **Supplementary Table S2**). Supplementary UV-B also tended to increase dry matter partitioning to stem, but the effect was not significant (**Figure 4B**; **Supplementary Table S2**).

### 3.3 Effects of supplemental radiation within and outside PAR on glandular trichome density and secondary metabolite biosynthesis

**Figure 5:**
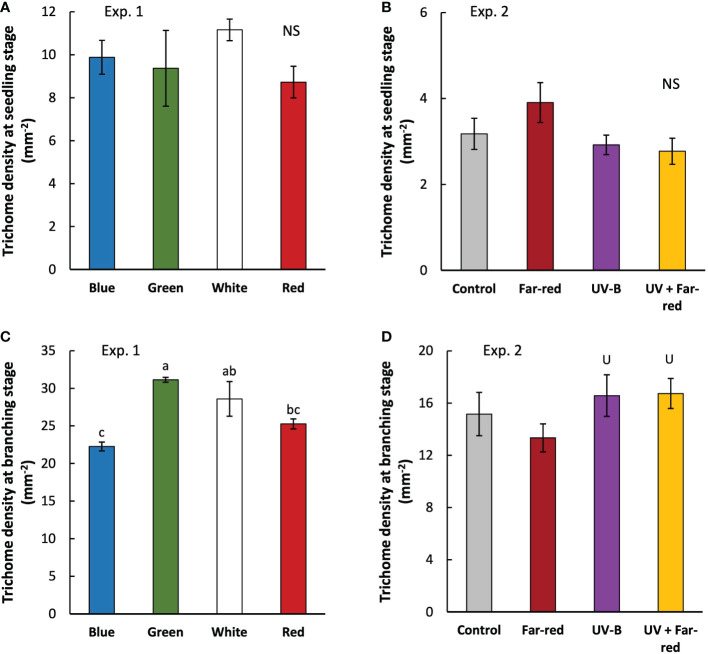
Trichome density measured at three weeks (seedling stage; **(A, B)** and five weeks (branching state; **(C, D)** after transplanting in treatments with supplemental radiation within (A, C; Exp. 1) and outside (B, D; Exp. 2) PAR wavelength range (values are mean ± s.e.; n = 4, with three plants in each statistical replicate). Details on the treatment abbreviations can be found in Figure 1. “NS” in panel A and B indicates non-significant treatment effects. Different letters in panel C indicate significant differences between treatments (*p* < 0.05; ANOVA test was done using log transformed data). “U” in panel D indicates significant effects from supplemental UV-B (*p* < 0.05).

At seedling stage, the colour of supplemental PAR hardly affected leaf trichome density, nor did the supplemental far-red or UV-B (**Figures 5A, B**). Trichome density was much higher in branching stage than in seedling stage, and treatment effects became significant **(Figure 5**). At branching stage, supplemental green resulted in the highest trichome density compared with other treatments, whereas supplemental blue resulted in the lowest trichome density (**Figure 5C**). Supplemental UV-B significantly increased trichome density at branching stage, whereas supplemental far-red did not have a significant effect (**Figure 5D**). There were no interactive effects between far-red and UV-B on trichome density.

Secondary metabolites, including dihydroartemisinic acid (DHAA), artemisinin, artemisinic acid (AA) and arteannuin B (AB), were hardly affected by the light treatments (**Table 1**; **Supplementary Tables S3**, **S4**). Leaf artemisinin concentration was generally higher in Exp.1 (0.2% ~ 0.4%) than in Exp. 2 (0.04% ~ 0.15%). In both experiments, AA concentration was relatively high, indicating a lack of photo-oxidative conversion to AB; in contrast, DHAA concentration was relatively low, indicating that most DHAA has been converted to artemisinin successfully. Artemisinin concentration was relatively low in the stem, especially in Exp. 2 that no artemisinin was found in the stem (**Supplementary Table S3**). Total amounts of secondary metabolites in the plant were not significantly affected by the light treatments (**Supplementary Table S4**).

**Table 1:** Leaf secondary metabolite concentrations (mg g^-1^ leaf dry weight) in treatments with supplemental blue, green, red and white (Exp. 1), and in treatments with supplemental far-red and ultraviolet-B (UV-B) (Exp. 2). Values are mean ± s.e. (n = 4, with three plants in each statistical replicate). None of the data showed significant treatment effects.

		Arteannuin B (AB)	Artemisinin	Dihydroartemisinic acid (DHAA)	Artemisinic acid (AA)
Exp. 1	Blue	0.032 ± 0.018	0.425 ± 0.412	0.053 ± 0.005	0.884 ± 0.236
	Green	0.033 ± 0.020	0.225 ± 0.181	0.052 ± 0.006	0.714 ± 0.088
	Red	0.029 ± 0.014	0.292 ± 0.277	0.063 ± 0.004	0.864 ± 0.158
	White	0.043 ± 0.015	0.320 ± 0.245	0.039 ± 0.005	0.802 ± 0.206
Exp. 2	Control	0.053 ± 0.009	0.149 ± 0.074	0.039 ± 0.010	0.321 ± 0.070
	Far-red	0.064 ± 0.003	0.144 ± 0.031	0.033 ± 0.009	0.309 ± 0.088
	UV-B	0.060 ± 0.006	0.121 ± 0.042	0.030 ± 0.004	0.231 ± 0.036
	Far-red + UV-B	0.059 ± 0.007	0.043 ± 0.028	0.037 ± 0.013	0.363 ± 0.143

## 4 Discussion

### 4.1 Supplemental green and UV-B increased leaf glandular trichome density

Artemisinin is biosynthesized in glandular trichomes of *A. annua*, which are commonly composed of 10 symmetrical cells (Olofsson et al., 2012). The biosynthetic pathway of artemisinin has been almost completely elucidated (Tang et al., 2014; Chen et al., 2017). In short, farnesyl diphosphate (FPP) is formed through mevalonate (MVA) pathway and 2-*C*-methyl-_D_-erythritol 4-phosphate (MEP) pathway, which is then converted into AA or DHAA *via* four trichome-specific enzymes (AaADS, AaCYP71AV1, AaDBR2 and AaALDH1) (Tang et al., 2014; Chen et al., 2017). DHAA is then transported to the trichome subcuticular space and converted to artemisinin *via* a photo-oxidative process (Brown and Sy, 2004). Studies have shown that overexpressing genes encoding trichome-related transcriptional factors (e.g. AaTAR2 and AaMIXTA1) increased artemisinin biosynthesis (Shi et al., 2018; Zhou et al., 2020), suggesting the potential of increasing trichome density for enhancing artemisinin production. Recently, increasing trichome density also has been considered as a new plant breeding strategy to enhance the yield of bioactive compounds for the pharmaceutical industry (Xiao et al., 2016). Here, we showed that trichome density could be increased by manipulating light conditions for *A. annua* growth, more specifically, by adding supplemental green or UV-B to background light in the greenhouse for *A. annua* production.

Trichome formation generally tends to increase in adverse environments to better cope with these conditions. This increase in trichome density may reduce transpiration rate, prevent from photodamage by reflecting sunlight, produce specialized secondary metabolites for defense responses to pests and pathogens, and absorb UV radiations to protect photosynthetic tissues (reviewed in Hauser, 2014). Many abiotic stresses – such as cold, heat, drought, salinity, heavy metal, and UV radiations – have been found to upregulate trichome initiation in different species (Filella and Peñuelas, 1999; Yan et al., 2012; Ning et al., 2016; Zhou et al., 2018; Zhang et al., 2019; Zheng et al., 2020). We also showed a positive effect of UV-B on trichome density in *A. annua* (**Figure 5D**). However, artemisinin concentration was not significantly affected by UV-B (**Table 1**), which is different from previous studies showing positive effects of UV radiations on artemisinin biosynthesis (Rai et al., 2011; Pan et al., 2014; Pandey and Pandey-Rai, 2015). Despite that many studies focus on trichome initiation responses to environmental factors including UV radiations, effects of other light wavelength than UV radiations have hardly received any attentions. Trichome initiation is known to be regulated by phytohormones especially jasmonates (JA) (reviewed in Chalvin et al., 2020), while JA signaling is further regulated by light signals (reviewed in Kazan and Manners, 2011; Ballaré, 2014). This suggests possible influences of light spectrum on trichome initiation *via* regulating JA signaling pathways.

Far-red is often found to involve in JA signaling pathways to regulate the production of secondary metabolites relevant with plant defense (Cerrudo et al., 2012; Leone et al., 2014), and potentially also interacts with UV-B (Mazza and Ballaré, 2015). However, we did not observe any significant effects of supplemental far-red on trichome initiation and artemisinin biosynthesis, nor any interactive effects between far-red and UV-B (**Figures 5A, B**; **Table 1**). Interestingly, we found a positive effect of supplemental green on trichome density (**Figure 5C**). To the best of our knowledge, we are the first to reveal the role of green light in trichome initiation, given that green light has long been considered as irrelevant for plant functioning as plants reflect relatively more irradiance in the wavelength range of green than other colors. Recently, green light has received more attentions, especially on regulating plant morphogenesis (Johkan et al., 2012; Schenkels et al., 2020; Zhang et al., 2021; Zhang et al., 2022), but few research focuses on secondary metabolites (reviewed in Landi et al., 2020). It seems that green light acts antagonistically to blue light, and reduces anthocyanin biosynthesis (Zhang and Folta, 2012). We found a negative effect of supplemental blue on trichome density (**Figure 5C**), suggesting an antagonistic role of green and blue in regulating trichome initiation given their opposite effects on trichome density. Nevertheless, artemisinin content was hardly affected by the color of supplemental radiation (**Table 1**). Generally, cryptochromes are proposed to be the receptor of green light, and senses green *via* FADH which is an intermediate form of fully oxidized chromophore excited by blue light (Kottke et al., 2006; Bouly et al., 2007; Liu et al., 2010; Sato et al., 2015). This is also relevant with the antagonistic effect of blue and green given that sensing of these two light signals is *via* the interconversion of flavin redox states of cryptochrome. Chico et al. (2014) showed that cryptochrome is involved in stabilizing the JA-related transcriptional factor MYC2. This could further affect any JA involved processes, including trichome initiation. Given the increasing interest in regulating trichome density in the pharmaceutical industry, more studies are needed to reveal the underlying mechanisms of light spectrum regulation on trichome initiation.

### 4.2 Supplemental green, UV-B and far-red decreased plant growth

In practise, the whole aerial part of *A. annua* is harvested as raw plant material for artemisinin production (Ferreira et al., 2005). Although plant artemisinin concentration heavily influences the price, the total payment is determined by the weight of the harvested plant materials (Ferreira et al., 2005). However, currently many studies have focused on increasing the artemisinin content in the plant, agronomic traits relevant with biomass production has been overlooked. Our results suggest that there was potentially a trade-off between increasing trichome density and biomass production, as supplemental green and UV-B, which were found to increase trichome density, decreased plant dry weight (**Figures 4**, **5C, D**). Despite that previous studies normally show increased artemisinin content by applying UV-B (Rai et al., 2011; Pan et al., 2014; Pandey and Pandey-Rai, 2015; Ma et al., 2020; Li et al., 2021), its effects on agronomic traits and plant biomass are less studied. In many plant species, UV-B is found to inhibit plant growth (reviewed in Yadav et al., 2020), but the effects of UV-B on artemisia growth are inconsistent. Pan et al. (2014) showed no effects of applying UV-B on plant biomass. Rai et al. (2011) found an increased biomass under UV-B treatment even with reduced leaf area; the increased number of mesophyll cells and spongy parenchyma found in the UV-B treated plants may suggest a higher leaf photosynthetic capacity, resulting in higher biomass. In our study, supplemental UV-B decreased plant dry weight, possibly because plant leaf area was reduced (due to shorter leaves on the main stem) (**Figure 2B**, **3D**), resulting in lower light interception and consequently less carbon assimilation. The inconsistency of UV-B effects on artemisia growth may be caused by the different intensity, duration and developmental stage of applying UV-B.

Green light may penetrate deeper inside the leaf tissue and in the plant canopy and may therefore enhance plant growth (Smith et al., 2017). Despite some studies showing a positive effect of green on biomass production (Kim et al., 2004; Schenkels et al., 2020) and others suggesting non-significant effects (Snowden et al., 2016; Zhang et al., 2021), we found that supplemental green decreased plant dry weight of *A. annua* (**Figure 4A**), which is possibly due to reduced leaf area (**Figure 2A**). Some studies suggested that green induced shade avoidance responses (Zhang et al., 2011; Schenkels et al., 2020). However, typical shade avoidance responses – such as increased internode length and steeper leaf elevation angle – were not found in *A. annua* grown under supplemental green (**Figures 3A–E**). Given the negative effects of supplemental green and UV-B on plant growth, their applications in greenhouse production of *A. annua* need critical evaluations to achieve a balance between increasing trichome density and maintaining biomass production.

Recently, there is an increasing interest of applying far-red in regulating plant growth and product quality in greenhouse crops. Adding supplemental far-red has been found to increase lettuce yield by increasing biomass partitioning to shoot to promote leaf area development (Jin et al., 2021), increase tomato yield by increasing fruit sink strength (Ji et al., 2020), and improve postharvest cold tolerance in tomato (Affandi et al., 2020). However, we found that in medicinal crop *A. annua*, far-red decreased plant growth (**Figure 4B**), likely due to the changes in the vertical distribution of leaf elevation angles (**Figure 3F**). The natural distribution of leaf angle in *A. annua* followed a very efficient pattern, i.e. leaf elevation angle gradually increased with increasing phytomer rank (**Figures 3E, F**). This results in more steeper leaf angle at the top, allowing more light penetration to the lower part of the plant, which is then captured by the more horizontally arranged leaves at the bottom. Supplemental far-red changed this leaf angle distribution by increasing angles of the lower leaves (**Figure 3F**), reducing the projected leaf area which potentially decreases light interception. Additionally, far-red reduced plant leaf area (**Figure 2B**), likely caused by the increase of biomass partitioning to stem (**Figure 4B**), which could potentially reduce plant light interception and carbon assimilation. We conclude that for greenhouse production of *A. annua*, supplemental far-red may not bring positive effects on plant growth.

### 4.3 Limitations and future perspectives

In general, plant artemisinin concentration is found to be the highest around flowering stage (Ferreira et al., 2005; Mannan et al., 2011; Rai et al., 2011). Short-day condition (a photoperiod of ~13 hours) is required for *A. annua* transitioning from vegetative to generative stage (Ferreira et al., 2005). Thus, flowering time – as well as harvest time – of field grown *A. annua* follows the seasonal changes of day length during the year. It is possible to provide short-day photoperiods in the greenhouse (even when natural daylength is long) to achieve all-year-round production. However, mostly this also means a smaller daily light integral, resulting in reductions in daily assimilation and final biomass. Therefore, although artemisinin concentration is higher in flowering stage (e.g. ~0.7%) than in vegetative stage (e.g. ~0.2%) (Rai et al., 2011), final yield of artemisinin may not necessarily be higher due to the reduction in biomass production. Our study only focused on vegetative plants grown under long-day conditions. Further studies are needed to evaluate the benefits between the relatively higher artemisinin concentration from short-day condition and more biomass growth from long-day condition for *A. annua* greenhouse production. Additionally, studies have shown that floral induction in chrysanthemum (a short-day species) can be induced under blue light extended long-day (Jeong et al., 2014; SharathKumar et al., 2021). It is worth to investigate the potential of using blue LEDs in *A. annua* production to induce flowering for higher artemisinin concentration while keeping long photoperiod for biomass production.

We hardly found any effects of light spectrum on artemisinin concentration, which is inconsistent with several previous studies (e.g. Rai et al., 2011; Pan et al., 2014; Zhang et al., 2018). However, it is worth to note that in our experiment, *A. annua* plants were grown in a greenhouse with high-pressure sodium (HPS) lamps installed, which are regular supplemental lights that are often used in commercial greenhouse production. These HPS lamps provided a light intensity that was much higher than the light intensity from the treatment LEDs. Therefore, the treatment LEDs with different colours worked more as a light signal instead of a direct resource for plant growth. Nevertheless, we found clear effects of treatment LEDs on plant morphology and trichome density, suggesting the importance of light signals in regulating artemisia photomorphogenesis and trichome formation. Given that the effects of light signals on artemisinin biosynthesis were not substantial, further studies are needed to investigate the effects of LEDs (providing different colours) with higher intensities to elucidate the effects of light as a direct resource for *A. annua* growth and secondary metabolism. Moreover, the underlying mechanisms of light regulations on expressions of key genes involved in artemisinin biosynthesis need to be clarified. These types of work could provide a comprehensive understanding and evaluation of supplemental lights on *A. annua* growth, development and secondary metabolism, which are needed before bringing *A. annua* into commercial greenhouse cultivation.

## 5 Conclusions

Bringing medicinal plant cultivation into the greenhouse has great potential to ensure stable supply and high quality of raw plant materials, and provide opportunities for manipulating growth conditions to enhance production of bioactive compounds. We attempted greenhouse production of *A. annua* and manipulated light environment for regulating artemisinin biosynthesis and plant growth. Supplemental green and UV-B increased leaf glandular trichome density, whereas plant growth was decreased possibly due to reduced leaf area. Supplemental far-red decreased plant growth, possibly due to increased leaf elevation angle in the lower leaves that reduced plant light capture efficiency. Artemisinin concentration was hardly affected by the spectrum of supplemental radiation. We conclude that there is a potential of manipulating supplemental radiation for increasing trichome density, however, the trade-off between increasing trichome density and plant growth needs to be considered. Furthermore, the underlying mechanisms of light spectrum regulation of artemisinin biosynthesis need to be clarified to further improve artemisinin biosynthesis for greenhouse production of *A. annua*.

## Data availability statement

The original contributions presented in the study are included in the article/**Supplementary Material**. Further inquiries can be directed to the corresponding authors.

## Author contributions

NZ and LM designed the research. HY, TH and HK conducted the measurements. NZ, HY and TH analysed the data. HK and LM secured funding. NZ drafted the manuscript and all co-authors made substantial contributions to improve the manuscript. All authors contributed to the article and approved the submitted version.
