# Supplementary material for: Towards greenhouse cultivation of Artemisia annua: The application of LEDs in regulating plant growth and secondary metabolism
Source: Front Plant Sci. 2023 Jan 18;13:1099713. doi: 10.3389/fpls.2022.1099713 (PMC9889874; doi:10.3389/fpls.2022.1099713)
Supplement: Supplementary file 1 [file DataSheet_1.docx]

Supplementary Material

# Supplementary Figures and Tables

## Supplementary Figures


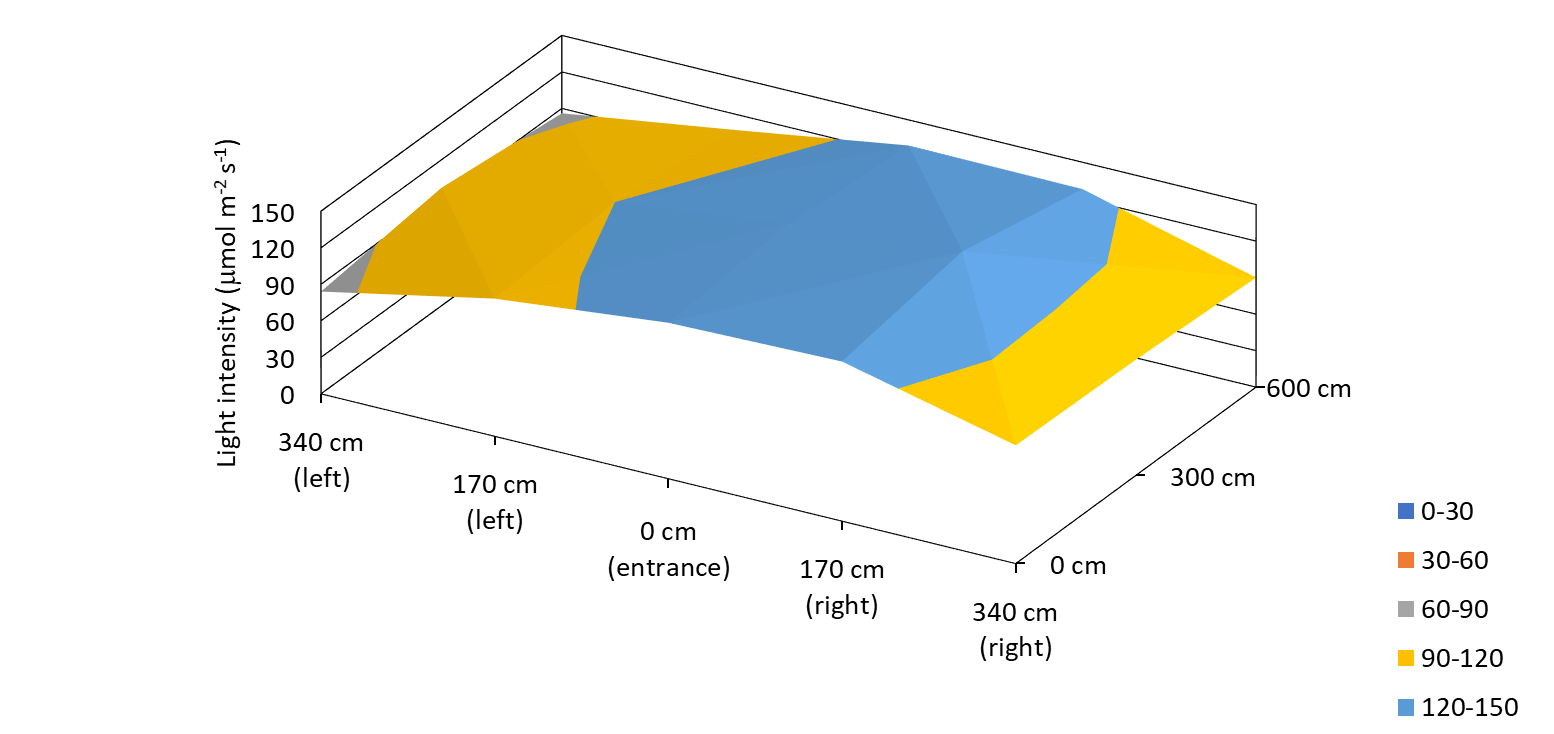


**Supplementary Figure 1.** Light distribution of the high-pressure sodium lamps in the greenhouse compartment (measured without solar light and treatment supplemental light).


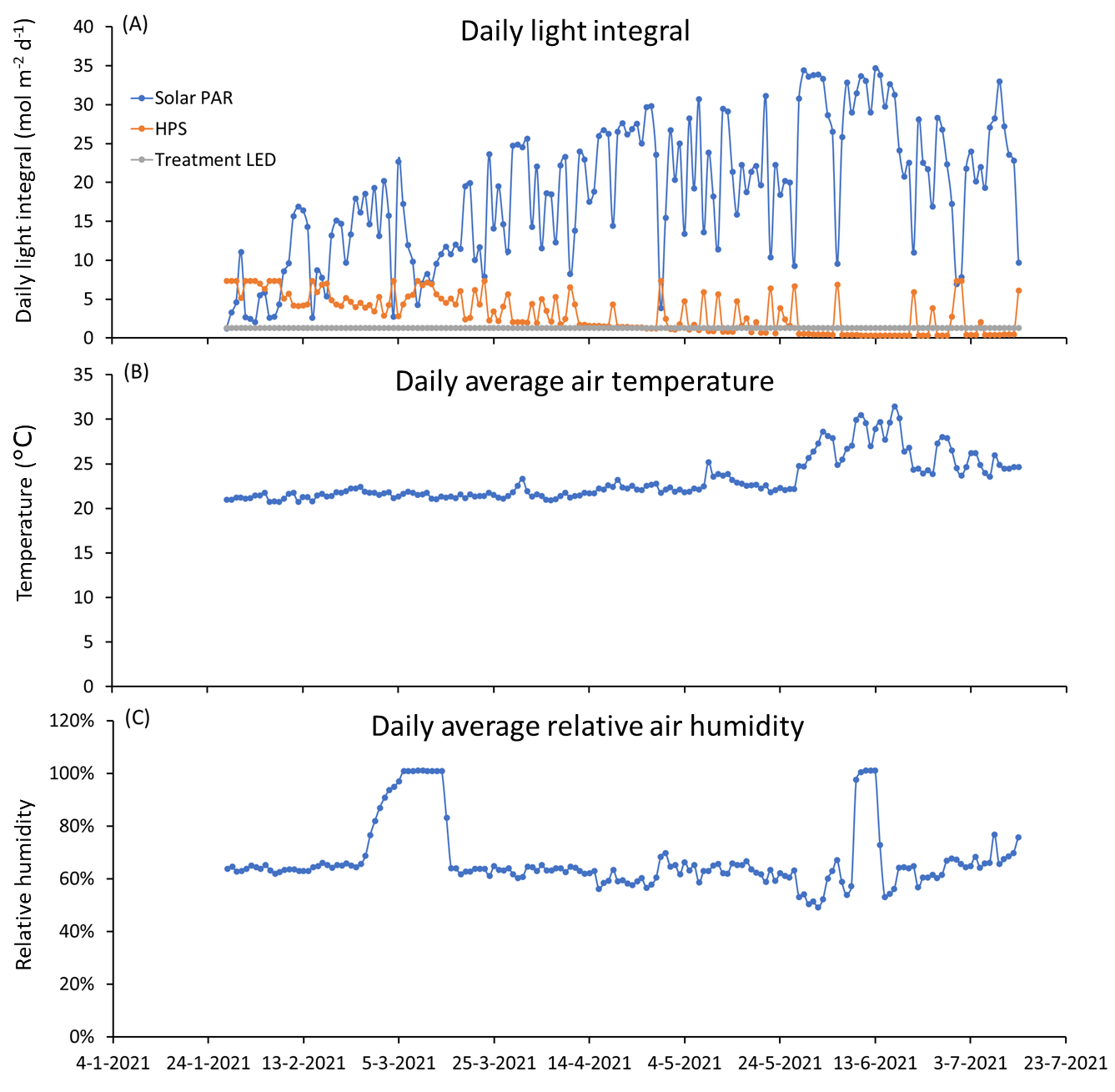


**Supplementary Figure 2.** Climate conditions during the experiment inside the greenhouse compartment, including (A) daily light integral of solar photosynthetically active radiation (PAR), high-pressure sodium (HPS) lamps and treatment LEDs, (B) daily average air temperature, and (C) daily average relative air humidity.


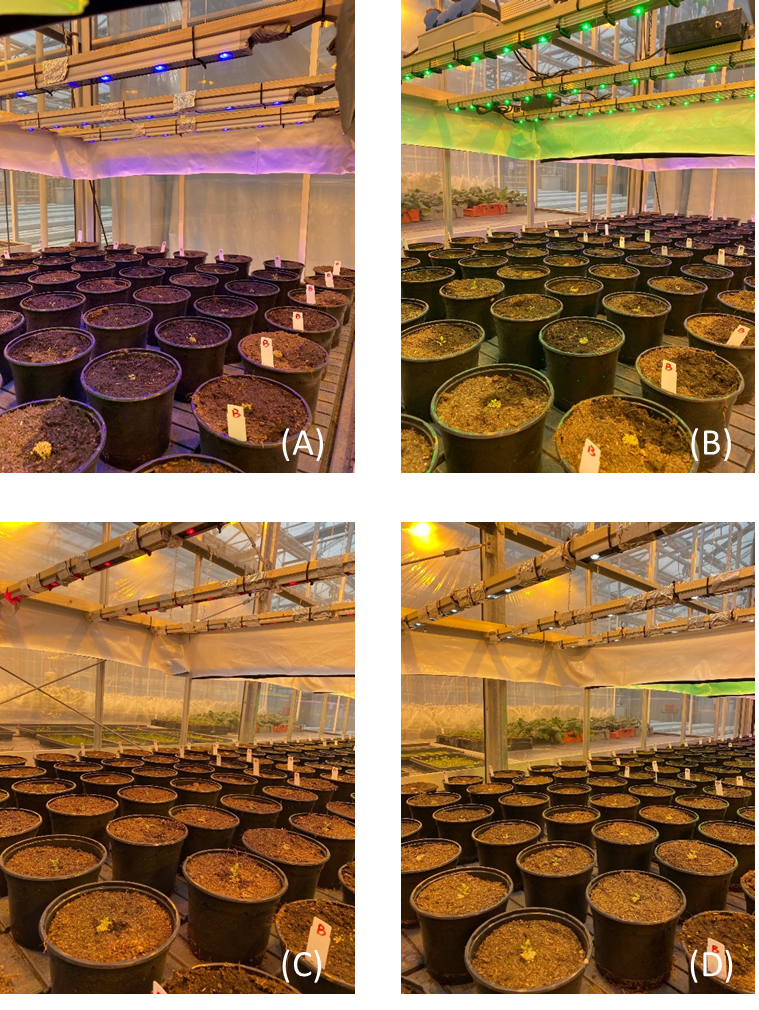


**Supplementary Figure 3.** Pictures of the light treatments in Exp. 1 with supplemental (A) blue, (B) green, (C) red and (D) white.


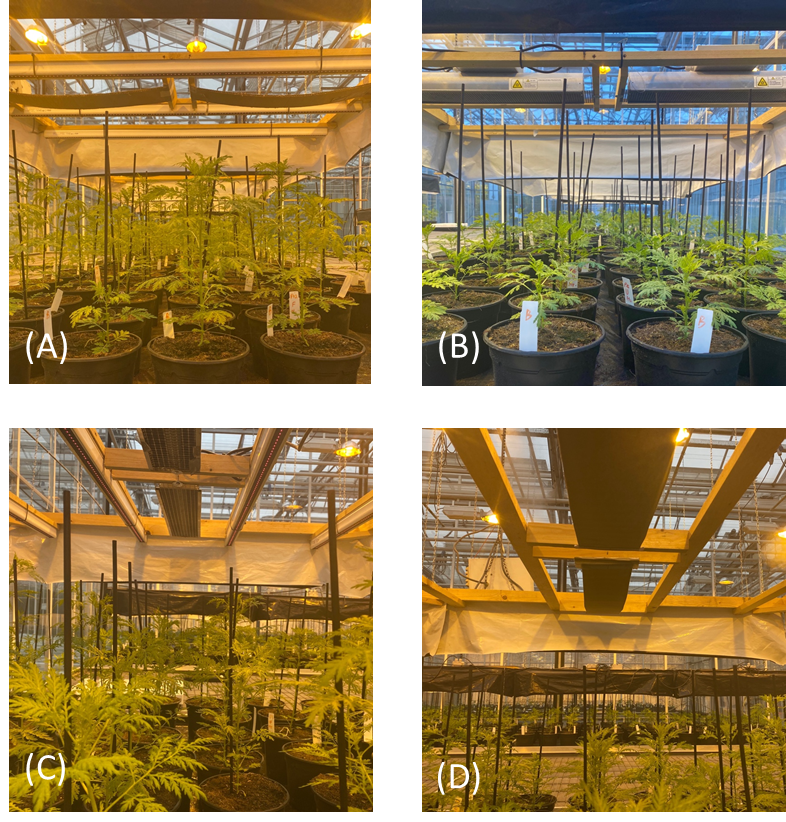


**Supplementary Figure 4.** Pictures of the four light treatments in Exp. 2 with supplemental (A) far-red, (B) UV-B and (C) far-red and UV-B, and (D) without supplemental LEDs.


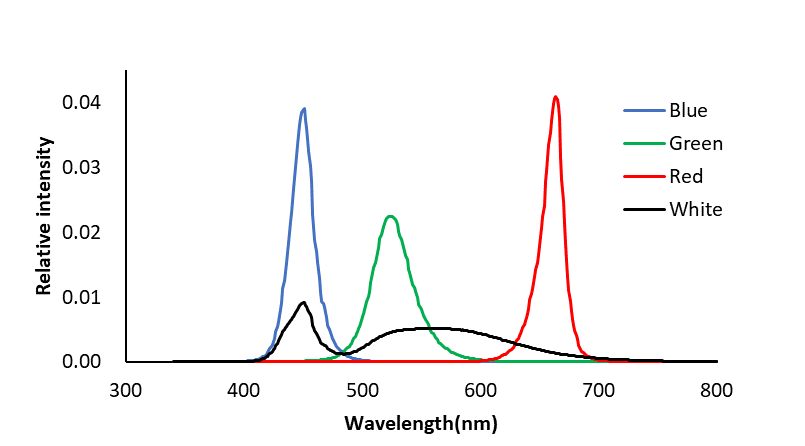


**Supplementary Figure 5.** Light spectrum of the blue, green, red and white LED modules used in Exp. 1. y-axis shows the proportion of light intensity at each wavelength that accounts for the total light intensity between 400-700 nm.


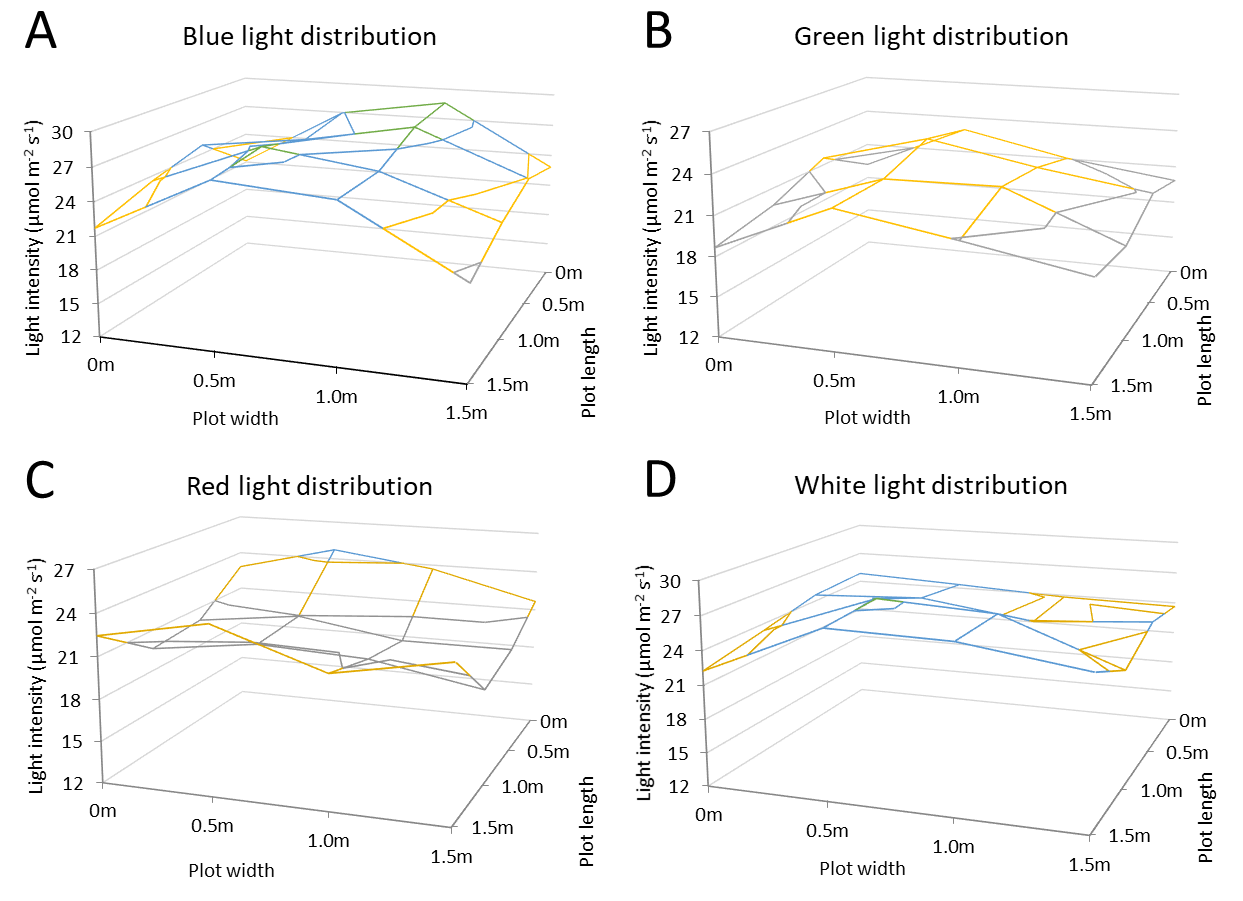


**Supplementary Figure 6.** Distribution of the irradiance from the LED frame in treatment with supplemental (A) blue, (B) green, (C) red and (D) white light in Exp. 1. Plot length and width represent the space of the plot.

**
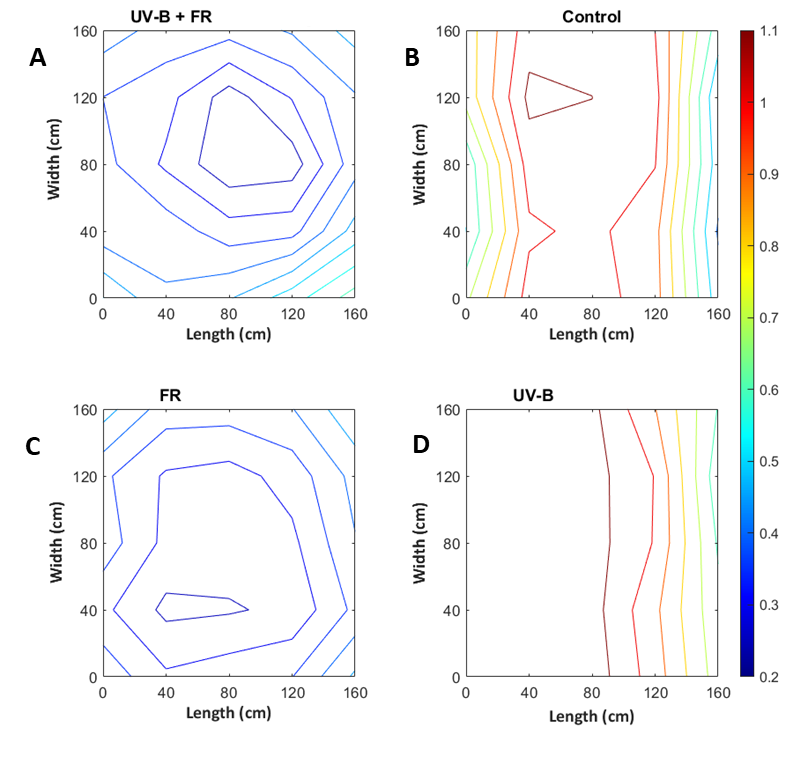
**

**Supplementary Figure 7.** Distribution of the red to far-red ratio in Exp. 2 in treatment (A) with supplemental far-red and UV-B, (B) without supplemental LEDs, (C) with supplemental far-red and (D) with supplemental UV-B.


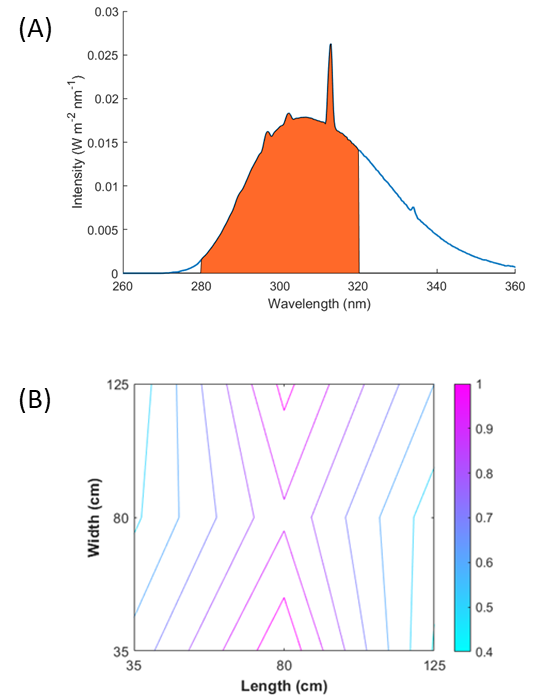


**Supplementary Figure 8.** (A) Light spectrum of the UV-B lamps measured in darkness. The orange area indicates the UV-B wavelength range. (B) Distribution of the UV-B light in a treatment plot.


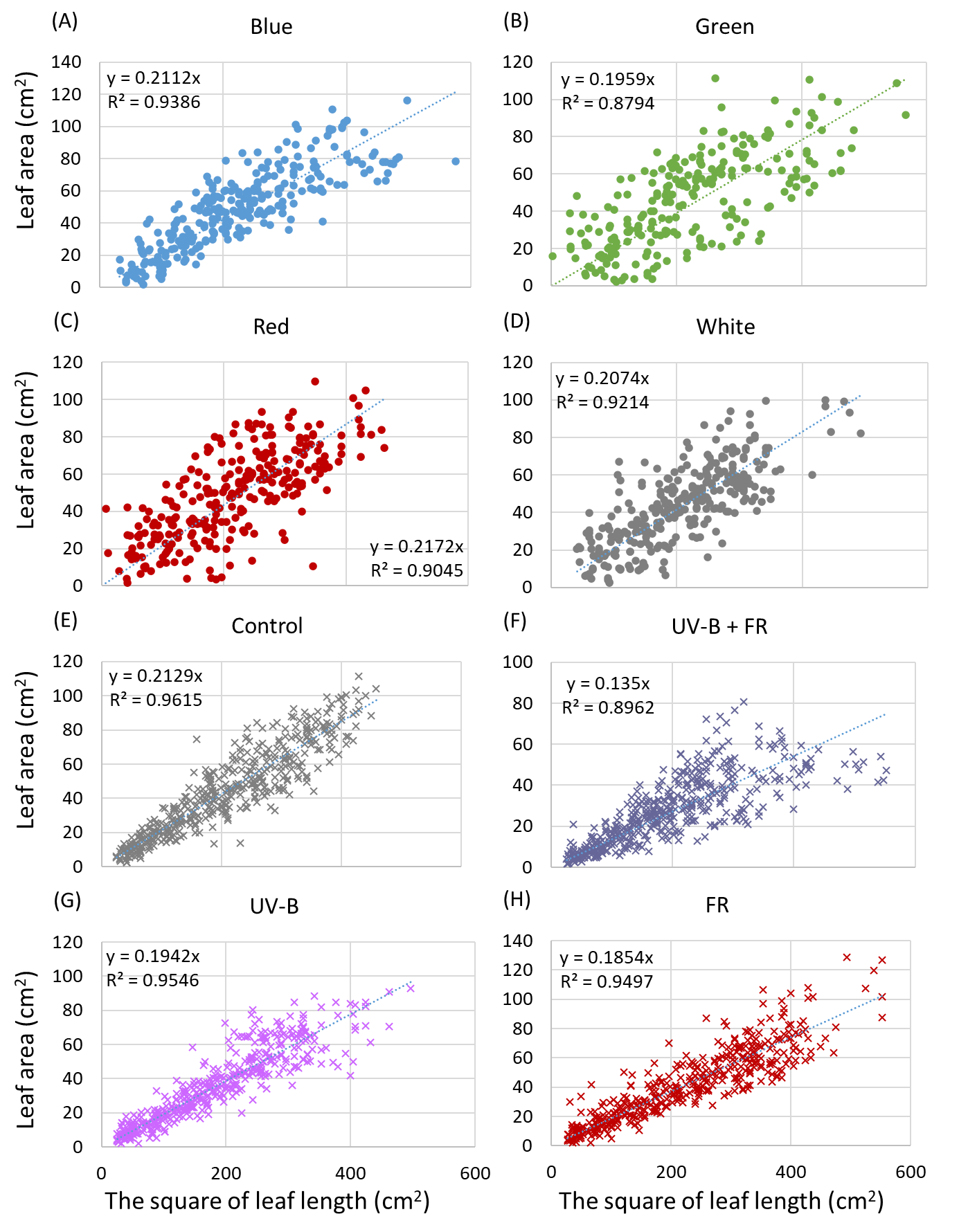


**Supplementary Figure 9.** The relationship between leaf length and leaf area from measured data in Exp. 1 in treatments with supplemental blue (A), supplemental green (B), supplemental red (C) and supplemental white (D), and from measured data in Exp. 2 in treatments without any supplemental radiation (E) and with supplemental UV-B and far-red (F), supplemental UV-B (G) and supplemental far-red (H).

## Supplementary Tables

Table S1. The transplanting date and harvest date in year 2021 (i.e. treatment duration) for each block of the two experiments.

|  |  | Block 1 | Block 2 | Block 3 | Block 4 |
| --- | --- | --- | --- | --- | --- |
| Exp. 1 | Transplanting | Jan 28^th^ | Feb 4^th^ | Mar 23^rd^ | Mar 26^th^ |
|  | Harvest | Mar 12^th^ | Mar 19^th^ | Apr 28^th^ | Apr 30^th^ |
| Exp. 2 | Transplanting | Mar 29^th^ | Apr 6^th^ | May 31^st^ | Jun 7^th^ |
|  | Harvest | May 4^th^ | May 11^th^ | Jul 6^th^ | Jul 13^th^ |

Table S2. Plant fresh and dry weights, and the fraction of dry matter partitioned to leaf and stem in treatments with supplemental blue, green, red and white (Exp. 1), as well as in treatments with supplemental far-red and ultraviolet-B (UV-B) (Exp. 2). Values are means (n = 4, with 15 plants in each statistical replicate). For Exp.1, different letters following the values indicate significant difference (*p* < 0.05). For Exp. 2, * represents a significant effect from far-red or UV-B, and “ns” means no significant effects (*p* < 0.05).

| Plant traits  Treatments | | Fresh weight (g plant^-1^) | | | Dry weight (g plant^-1^) | | | Dry matter partitioning (%) | |
| --- | --- | --- | --- | --- | --- | --- | --- | --- | --- |
|  |  | Leaf | Stem | Plant total | Leaf | Stem | Plant total | Leaf | Stem |
| Exp. 1 | Blue | 22.6 ab | 24.4 a | 47.0 a | 2.7 a | 2.1 a | 4.8 a | 57 b | 43 a |
|  | Green | 21.7 b | 21.9 b | 43.6 b | 2.6 b | 1.9 b | 4.4 b | 59 a | 41b |
|  | Red | 25.2 a | 26.1 a | 51.3 a | 2.9 a | 2.1 a | 5.0 a | 59 ab | 41 ab |
|  | White | 24.2 a | 26.9 a | 51.1 a | 2.9 a | 2.2 a | 5.2 a | 57 b | 43 a |
|  | SEE | 5.5 | 7.7 | 12.2 | 0.7 | 0.7 | 1.3 | 4 | 4 |
| Exp. 2 | Control | 22.9 | 26.7 | 49.5 | 2.5 | 2.0 | 4.5 | 57 | 43 |
|  | Far-red | 16.1 | 25.0 | 41.1 | 2.1 | 2.2 | 4.2 | 50 | 50 |
|  | UV-B | 21.3 | 27.5 | 47.4 | 2.3 | 2.0 | 4.2 | 54 | 46 |
|  | Far-red + UV-B | 14.2 | 23.6 | 37.7 | 1.8 | 2.0 | 3.8 | 49 | 51 |
|  | SEE | 5.4 | 9.1 | 12.6 | 0.6 | 0.7 | 1.3 | 10 | 10 |
| Effects of far-red | | * | * | * | * | ns | * | * | * |
| Effects of UV-B | | * | ns | ns | * | ns | * | ns | ns |
| Interactive effects of far-red and UV-B | | ns | ns | ns | ns | ns | ns | ns | ns |

Table S3. Stem secondary metabolite concentrations (mg g^-1^ stem dry weight). Values are mean ± s.e. (n = 4, with three plants in each statistical replicate).

|  | | Arteannuin B | Artemisinin | Dihydroartemisinic acid | Artemisinic acid |
| --- | --- | --- | --- | --- | --- |
| Exp. 1 | Blue | 0.0031 ± 0.0018 | 0.0172 ± 0.0123 | 0.0003 ± 0.0003 | 0.0219 ± 0.0083 |
|  | Green | 0.0043 ± 0.0026 | 0.0041 ± 0.0041 | 0.0016 ± 0.001 | 0.0285 ± 0.0096 |
|  | Red | 0.0045 ± 0.0029 | 0.0026 ± 0.0017 | 0.0008 ± 0.0008 | 0.0303 ± 0.0034 |
|  | White | 0.0012 ± 0.0012 | 0.0359 ± 0.0236 | 0 | 0.0549 ± 0.0283 |
| Exp. 2 | Control | 0.0153 ± 0.0036 | 0 | 0.0044 ± 0.0033 | 0.0973 ± 0.0321 |
|  | Far-red | 0.0116 ± 0.0041 | 0 | 0.0033 ± 0.0027 | 0.0834 ± 0.0296 |
|  | UV-B | 0.0110 ± 0.0025 | 0 | 0.0033 ± 0.0022 | 0.0694 ± 0.0229 |
|  | Far-red + UV-B | 0.0150 ± 0.0018 | 0 | 0.0026 ± 0.0016 | 0.0683 ± 0.0187 |

Table S4. Total amount of secondary metabolites in the plant (mg plant^-1^, including metabolites measured in both leaves and stem) in treatments with supplemental blue, green, red and white (Exp. 1), as well as in treatments with supplemental far-red and ultraviolet-B (UV-B) (Exp. 2). Values are mean ± s.e. (n = 4, with three plants in each statistical replicate).

|  | | Arteannuin B | Artemisinin | Dihydroartemisinic acid | Artemisinic acid |
| --- | --- | --- | --- | --- | --- |
| Exp. 1 | Blue | 0.105 ± 0.061 | 1.082 ± 1.044 | 0.142 ± 0.013 | 2.301 ± 0.486 |
|  | Green | 0.090 ± 0.053 | 0.692 ± 0.607 | 0.133 ± 0.015 | 1.868 ± 0.278 |
|  | Red | 0.095 ± 0.047 | 0.912 ± 0.871 | 0.184 ± 0.004 | 2.536 ± 0.380 |
|  | White | 0.136 ± 0.049 | 0.981 ± 0.762 | 0.114 ± 0.018 | 2.382 ± 0.529 |
| Exp. 2 | Control | 0.161 ± 0.028 | 0.365 ± 0.165 | 0.102 ± 0.026 | 0.962 ± 0.204 |
|  | Far-red | 0.157 ± 0.010 | 0.296 ± 0.061 | 0.075 ± 0.022 | 0.802 ± 0.225 |
|  | UV-B | 0.162 ± 0.029 | 0.276 ± 0.102 | 0.071 ± 0.007 | 0.633 ± 0.057 |
|  | Far-red + UV-B | 0.138 ± 0.018 | 0.080 ± 0.049 | 0.070 ± 0.022 | 0.768 ± 0.242 |
